# Métastases au niveau des sites d’introduction des trocarts secondaires à un adénocarcinome de la vésicule biliaire

**DOI:** 10.11604/pamj.2018.31.52.14630

**Published:** 2018-09-24

**Authors:** Mohamed Essarghini, Ahmed Bounaim

**Affiliations:** 1 Service de Chirurgie Viscérale et Digestive de l’Hôpital Militaire d’Instruction Mohamed V de Rabat, Maroc

**Keywords:** Adénocarcinome de la vésicule biliaire, métastases de la paroi abdominale, laparoscopie, Gallbladder, adenocarcinoma, metastases, abdominal wall, laparoscopy

## Abstract

Fortuitous systematic histological examination discovery of gallbladder adenocarcinoma after laparoscopic cholecystectomy performed due to acute cholecystitis is rare. It requires reoperation through subcostal approach for vesicular bed resection as well as excision of the trocar trajectories. We here report the case of a 73-year old female patient with a history of laparoscopic surgery for acute cholecystitis performed three years before. Anatomopathological examination had showed tubulo-papillary adenocarcinoma of the vesicle, but the patient refused reoperation. Currently, she presented with two masses in the anterior abdominal wall, one of which was ulcerated. Abdominal CT scan objectified two parietal and peritoneal tissue masses and CA19-9 dosage was largely positive at 946 U/ml (30 times the normal value). Biopsy under local anesthesia confirmed tumor recurrence and the patient was referred to the Department of Oncology for GEMOX chemotherapy before revaluation for possible surgery.

## Image en médecine

La découverte fortuite de l’adénocarcinome de la vésicule biliaire après cholécystectomie cœlioscopique pour cholécystite aiguë à travers l’examen histologique systématique est rare. Cette situation impose une reprise chirurgicale par voie sous costale pour résection du lit vésiculaire ainsi que des trajets d’introduction des trocarts. Nous présentons un cas d’une patiente de 73 ans qui a été opérée il y a trois ans pour cholécystite aiguë par voie laparoscopique et dont l’examen anatomopathologique a révélé un adénocarcinome tubulo-papillaire de la vésicule mais la patiente a refusé la réintervention. Actuellement, elle consulte pour deux masses de la paroi abdominale antérieure dont une ulcérée. Une tomodensitométrie abdominale a objectivé deux masses tissulaires péritonéo-parietales et le dosage du CA19-9 est largement positif à 946 U/ml (soit 30 fois la valeur normale). Une biopsie sous anesthésie locale a confirmé la récidive tumorale et la patiente a été adressée en oncologie pour chimiothérapie type GEMOX avant réévaluation pour éventuelle chirurgie.

**Figure 1:**
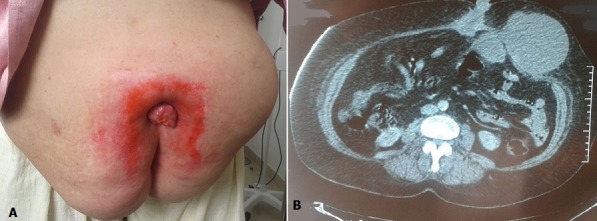
A) tumeur bourgeonnante et ulcérée de l’ombilic avec masse de la paroi abdominale antérieure sur les cicatrices des orifices des trocarts; B) image scannographique montrant des lésions péritonéo-pariétales; ombilicale de 62 sur 37 mm et au niveau de la paroi abdominale antérolatérale gauche de 106 sur 84 mm

